# Direct versus indirect epiglottis elevation in cervical spine movement during videolaryngoscopic intubation under manual in-line stabilization: a randomized controlled trial

**DOI:** 10.1186/s12871-023-02259-x

**Published:** 2023-09-07

**Authors:** Seungeun Choi, Dong Ju Lee, Kyung Won Shin, Yoon Jung Kim, Hee-Pyoung Park, Hyongmin Oh

**Affiliations:** Department of Anesthesiology and Pain Medicine, Seoul National University Hospital, Seoul National University College of Medicine, 101, Daehak-ro, Jongno-gu, Seoul, Republic of Korea

**Keywords:** Cervical spine movement, Videolaryngoscopic intubation, Glottis exposure method, Direct epiglottis elevation, Indirect epiglottis elevation

## Abstract

**Background:**

During videolaryngoscopic intubation, direct epiglottis elevation provides a higher percentage of glottic opening score than indirect epiglottis elevation. In this randomized controlled trial, we compared cervical spine movement during videolaryngoscopic intubation under manual in-line stabilization between the two glottis exposure methods.

**Methods:**

Videolaryngoscopic intubation under manual in-line stabilization was performed using C-MAC® D-blade: direct (n = 51) and indirect (n = 51) epiglottis elevation groups. The percentage of glottic opening score was set equally at 50% during videolaryngoscopic intubation in both groups. The primary outcome measure was cervical spine movement during videolaryngoscopic intubation at the occiput–C1, C1–C2, and C2–C5. The secondary outcome measures included intubation performance (intubation success rate and intubation time).

**Results:**

Cervical spine movement during videolaryngoscopic intubation was significantly smaller at the occiput–C1 in the direct epiglottis elevation group than in the indirect epiglottis elevation group (mean [standard deviation] 3.9 [4.0] vs. 5.8 [3.4] °, P = 0.011), whereas it was not significantly different at the C1–C2 and C2–C5 between the two groups. All intubations were successful on the first attempt, achieving a percentage of glottic opening score of 50% in both groups. Intubation time was longer in the direct epiglottis elevation group (median [interquartile range] 29.0 [24.0–35.0] vs. 22.0 [18.0–27.0] s, P < 0.001).

**Conclusions:**

When performing videolaryngoscopic intubation under manual in-line stabilization, direct epiglottis elevation can be more beneficial than indirect epiglottis elevation in reducing cervical spine movement during videolaryngoscopic intubation at the occiput–C1.

**Trial registration:**

Clinical Research Information Service (number: KCT0006239, date: 10/06/2021).

## Introduction

Excessive cervical spine movement during tracheal intubation, can put the patient at risk for detrimental consequences such as spinal cord injury, particularly in patients with cervical spine instability [1–3]. To stabilize the cervical spine in such patients, it is recommended to apply manual in-line stabilization throughout tracheal intubation [4]. Regardless of airway difficulty or cervical spine stabilization, videolaryngoscopy offers several advantages over direct laryngoscopy for both patients and manikins, such as a better laryngeal view, ease of tracheal intubation, and smaller cervical spine movement during intubation [5–14]. However, cervical spine movement, to a certain extent, is inevitable, even during videolaryngoscopic intubation under cervical spine stabilization, because a lifting force must be applied to visualize the glottis [15–19]. Therefore, finding additional methods to reduce cervical spine movement during videolaryngoscopic intubation under cervical spine stabilization has clinical significance.

In general, the blade tip is placed in the vallecula, and the epiglottis is elevated indirectly during laryngoscopic intubation. When such indirect epiglottis elevation yields a poor glottic view, direct epiglottis elevation, which lifts the epiglottis directly with the blade tip, can be used [20]. Indeed, a prospective study showed significant improvement in glottis exposure during videolaryngoscopic intubation with direct epiglottis elevation compared to indirect epiglottis elevation [21]. Therefore, we speculated that direct epiglottis elevation may require less lifting force, resulting in smaller cervical spine movement during videolaryngoscopic intubation to obtain the same level of glottis exposure than indirect epiglottis elevation, even under cervical spine stabilization [15]. However, no study has compared cervical spine movement during videolaryngoscopic intubation according to the glottis exposure method.

We hypothesized that direct epiglottis elevation would result in smaller cervical spine movement during videolaryngoscopic intubation under manual in-line stabilization than indirect epiglottis elevation. In this study, cervical spine movement during videolaryngoscopic intubation was compared between direct epiglottis elevation and indirect epiglottis elevation in patients receiving videolaryngoscopic intubation under manual in-line stabilization. In addition, intubation performance and intubation-related airway complications were assessed in the two glottis exposure methods in such patients.

## Methods

### Ethics

This randomized controlled trial was approved by the Institutional Review Board of Seoul National University Hospital (number: 2104-019-1210, date: 28/05/2021, study duration: 28/06/2021–04/02/2022) and was registered at the Clinical Research Information Service (number: KCT0006239, date: 10/06/2021) prior to patient enrollment. Written informed consent was obtained from all patients prior to participation in this study. This study was performed in compliance with the Declaration of Helsinki., and this paper was written in accordance with the applicable Consolidated Standard of Reporting Trials guidelines.

### Population

Patients aged 20–65 years who were scheduled for elective neurointervention under general anesthesia at Seoul National University Hospital were included in this study. Patients with a lesion on the upper airway or cervical spine, a history of surgery or radiotherapy on the upper airway or cervical spine, a high risk for aspiration (gastroesophageal reflux disease and inadequate fasting time) and dental injury (loose teeth), or coagulopathy were excluded from this study.

### Randomization

An anesthesiologist unrelated to this study made a random allocation sequence with four- and six-sized blocks using software (Random Allocation Software version 1.0.0; Isfahan University of Medical Sciences, Isfahan, Iran). Based on the random allocation sequence, patients were assigned to two parallel groups (direct and indirect epiglottis elevation groups) in a 1:1 ratio. The random allocation sequence was stored in a sealed opaque envelope, and the group assignment was conducted just before anesthetic induction by a nurse unrelated to this study.

### Protocol

Patients entered the intervention room without premedication. Airway-related parameters (modified Mallampati class, interincisor gap, thyromental distance, sternomental distance, and neck circumference) were evaluated in the sitting position. Then the patients were monitored with electrocardiography, pulse oximetry, and non-invasive blood pressure measurement and the patient’s head was placed on a pillow of 5 cm high in the supine position. After sufficient preoxygenation, anesthesia was induced with target-controlled infusion of remifentanil (effect site concentration: 4 ng/ml) and a bolus injection of propofol (1.5–2 mg/kg). After confirming loss of consciousness, mask ventilation was performed with 1.5 vol% of sevoflurane and rocuronium (0.6–0.8 mg/kg) was administered to facilitate videolaryngoscopic intubation. To minimize hemodynamic responses by videolaryngoscopic intubation, topical anesthesia on the anterior and posterior surfaces of the epiglottis and laryngeal inlet was performed with 10% lidocaine spray under videolaryngoscopic aid. After removing the videolaryngoscope from the oral cavity, the patient’s head and neck were placed in the neutral position on the same pillow. Manual in-line stabilization was applied and maintained by another anesthesiologist, who were blinded to the group assignment, until successful intubation was confirmed by waveform capnography. This anesthesiologist was at the side of the anesthesiologist performing videolaryngoscopic intubation and held the patient’s head firmly, including the mastoid process, applying sufficient force to minimize movement of the patient’s head and neck [22].

All orotracheal intubations were performed by one skilled anesthesiologist with accumulated experience of more than 100 successful videolaryngoscopic intubations after confirming train-of-four count of 0. A videolaryngoscope with a hyperangulated blade (C-MAC® D-blade; Karl Storz, Tuttlingen, Germany) was used for intubation. A plain tracheal tube (Covidien™ Shiley™ Hi-Lo oral/nasal tracheal tube; Medtronic, Minneapolis, MN, USA; internal diameter: 7.5 mm for males and 7.0 mm for females) was mounted on a malleable stylet with 60° angulation at the proximal edge of the cuff. A lateral cervical spine radiograph was taken before intubation attempt using a biplanar angiographic unit (Integris Allura™; Philips, Amsterdam, Netherlands).

**Fig. 1:**
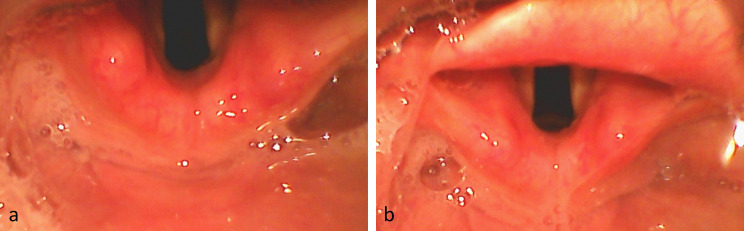
Glottis exposure during videolaryngoscopic intubation through direct **(a)** and indirect **(b)** epiglottis elevations

During videolaryngoscopic intubation, the blade tip was placed in the vallecula to lift the epiglottis indirectly and under the epiglottis to lift the epiglottis directly in the indirect and direct epiglottis elevation groups, respectively (Fig. 1). In both groups, the videolaryngoscope was manipulated as gently as possible to achieve a percentage of glottic opening score of 50%. In other words, the lifting force was delicately adjusted to make the same degree of glottis exposure. If a percentage of glottic opening score of 50% could not be met, despite the utmost effort, the highest possible percentage of glottic opening score was obtained and recorded. Airway maneuvers, such as external laryngeal manipulation and jaw thrust, were not applied even when the percentage of glottic opening score did not reach 50%, to avoid their potential effects on cervical spine movement during videolaryngoscopic intubation. The insertion of the tracheal tube was paused just after its tip was placed on the glottis, and the lateral cervical spine radiograph was taken again. Then the stylet was removed, and the tracheal tube was advanced into the trachea. Intubation time, which was defined as the time interval between oral insertion of the videolaryngoscope and placement of the tube tip on the glottis, was measured.

A failed intubation attempt was defined as an intubation time exceeding 3 min or a peripheral oxygen saturation lowered to less than 90%. If an intubation attempt was unsuccessful, the study protocol called for rescue mask ventilation for more than 1 min before the next intubation attempt. To minimize dropouts, a maximum of three consecutive intubation attempts would be permitted before being recorded as an intubation failure. In case of intubation failure, orotracheal intubation would be attempted again after removing the applied manual in-line stabilization or using another intubation device. In case of hemodynamic instability requiring immediate management during intubation attempt, the intubation attempt would be discontinued and the case would be excluded from data analysis.

At the time of extubation, blood in the oral cavity and blood staining on the tracheal tube were checked. In addition, hoarseness and sore throat were evaluated at 1 and 24 h after intervention. The severity of sore throat was assessed using the numeric rating scale (0 for no pain and 10 for the worst imaginable pain).

### Measurement of cervical spine angles

**Fig. 2:**
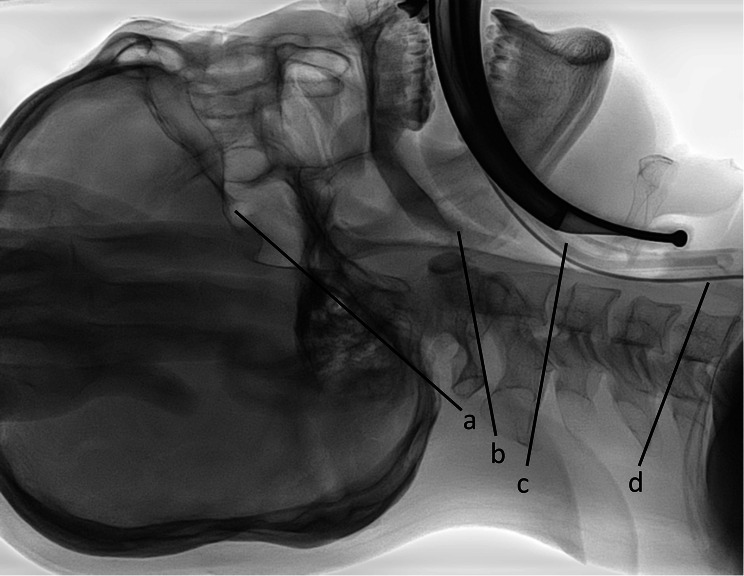
Reference lines of the occiput **(a)**, C1 **(b)**, C2 **(c)** and C5 **(d)**. **a**: the line connecting the sellar base and the opisthion. **b**: the line connecting the inferior cortical margin of C1 anterior arch and that of the C1 spinous process. **c**: the line connecting anteroinferior cortical margin of the C2 body and the inferior cortical margin of the C2 spinous process. **d**: the line parallel to the superior endplate of the C5 body

All lateral cervical spine radiographs taken before and during videolaryngoscopic intubation were archived and analyzed in the Picture Archiving and Communication System (IFINITT PACS version 5.0.0.143, Infinitt Healthcare, Seoul, Korea). The reference lines of the occiput, C1, C2, and C5 were defined as the line connecting the sellar base and the opisthion, the line connecting the inferior cortical margin of the C1 anterior arch and that of the C1 spinous process, the line connecting the anteroinferior cortical margin of the C2 body and the inferior cortical margin of the C2 spinous process, and the line parallel to the superior endplate of the C5 body, respectively (Fig. 2) [23–25]. A total of three cervical spine angles of the occiput–C1, C1–C2, and C2–C5 were measured at the intersection of the reference lines by an anesthesiologist who was blinded to the study protocol and group assignment.

### Outcomes

The primary outcome measure was cervical spine movement during videolaryngoscopic intubation, which was obtained by subtracting cervical spine angle before videolaryngoscopic intubation from that during videolaryngoscopic intubation, at the occiput–C1, C1–C2, and C2–C5. The secondary outcome measures were intubation performance (intubation success rate, number of intubation attempts, intubation time, and percentage of glottic opening score) and intubation-related airway complications (bleeding, hoarseness, and sore throat).

### Sample size calculation

In a randomized crossover study on orotracheal intubation under cervical spine stabilization using C-MAC® D-blade, the mean and standard deviation of cervical spine movement during videolaryngoscopic intubation through indirect epiglottis elevation at the occiput–C1 were 6.8° and 5.0°, respectively [24]. Assuming that 50% reduction in this cervical spine movement by direct epiglottis elevation is clinically significant, at least 102 patients (51 patients in each group) were required based on normal approximation using the Z statistic with α, β, and a dropout rate of 0.017 (0.5/3), 0.2, and 10%, respectively.

### Statistical analysis

Software (SPSS version 25; IBM Corp., Armonk, NY, USA) was used for all statistical analyses. For continuous variables, the normality of their data distribution was first assessed using Shapiro–Wilk test and evaluated once again using histograms. The normally and non-normally distributed variables are presented as mean (standard deviation) and median (interquartile range), and were compared using Student’s t-test and Mann–Whitney U-test, respectively. To calculate median differences for non-normally distributed variables, Hodges–Lehmann estimation was used. Categorical variables are presented as number (proportion) and were compared using Fisher’s exact test or Pearson’s chi-square test if > 20% and ≤ 20% of cells had expected count less than 5, respectively. For demographic and airway-related variables, standardized differences were calculated to determine whether there were variables with significant imbalance between the direct and indirect epiglottis elevation groups; a 95% confidence interval (CI) for a standardized difference, which did not contain 0, was considered significantly imbalanced. If there were such variables, multiple linear regression analysis was performed using a stepwise method to adjust their effects on the primary outcome measure. For the primary outcome measure, Student t-test was used and Bonferroni correction was applied to compensate for multiple comparisons; a P value less than 0.017 (0.05/3) was considered statistically significant. For the secondary outcome measures, a P value less than 0.05 was assumed to be statistically significant.

## Results

**Fig. 3:**
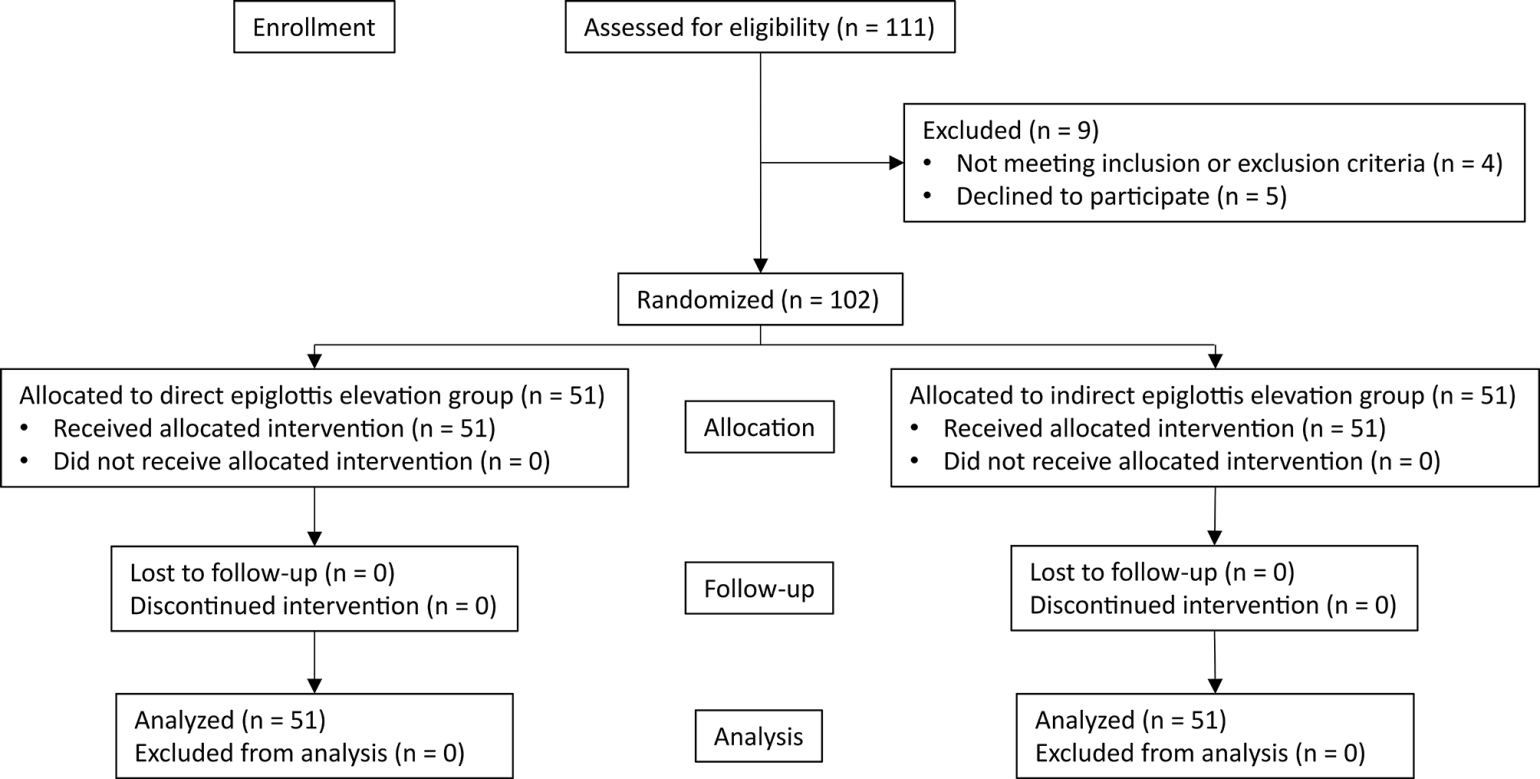
Consolidated Standards of Reporting Trials flow diagram

A total of 102 patients were enrolled in this study between June 2021 and February 2022 (Fig. 3). Without any dropout in this study, all enrolled patients were included in the analysis. There were no significant imbalances in demographic or airway-related variables between the direct and indirect epiglottis elevation groups, except for fewer male (10 [19.6%] vs. 20 [39.2%], standardized difference in proportions [95% CI] − 0.44 [− 0.83, − 0.05]), shorter interincisor gap (4.0 [4.0–5.0]) vs. 4.5 [4.0–5.0] cm, standardized difference in means [95% CI] − 0.46 [− 0.86, − 0.07]), and shorter thyromental distance (8.0 [8.0–9.0]) vs. 9.0 [8.0–9.5]) cm, standardized difference in means [95% CI] − 0.43 [− 0.82, − 0.03]) in the direct epiglottis elevation group (Table 1).

**Table 1:** Comparison of demographic, airway-related, and anesthetic variables between direct and indirect epiglottis elevation groups

Variable	Direct epiglottis elevation group (n = 51)	Indirect epiglottis elevation group (n = 51)	Standardized difference* (95% CI)
Demographics			
Age (y)	56.0 (52.0–61.0)	57.0 (52.0–62.0)	−0.09 (− 0.48, 0.30)
Male sex	10 (19.6%)	20 (39.2%)	−0.44 (− 0.83, − 0.05)
Height (cm)	159.2 (7.8)	161.0 (8.3)	−0.23 (− 0.62, 0.16)
Weight (kg)	61.7 (9.9)	62.1 (10.9)	−0.04 (− 0.43, 0.34)
BMI (kg/m^2^)	23.7 (22.2–26.2)	23.3 (21.6–26.6)	0.13 (− 0.26, 0.52)
Airway			
Modified Mallampati class			0.16 (− 0.22, 0.55)
1	14 (27.5%)	23 (45.1%)	
2	18 (35.3%)	11 (21.6%)	
3	12 (23.5%)	8 (15.7%)	
4	7 (13.7%)	9 (17.6%)	
Interincisor gap (cm)	4.0 (4.0–5.0)	4.5 (4.0–5.0)	−0.46 (− 0.86, − 0.07)
Thyromental distance (cm)	8.0 (8.0–9.0)	9.0 (8.0–9.5)	−0.43 (− 0.82, − 0.03)
Sternomental distance (cm)	16.0 (15.0–17.0)	16.0 (15.0–17.5)	−0.32 (− 0.72, 0.07)
Neck circumference (cm)	35.0 (33.0–37.0)	35.0 (33.0–38.0)	−0.09 (− 0.47, 0.30)
Anesthesia			
ASA physical status			0.03 (− 0.36, 0.42)
1	20 (39.2%)	22 (43.1%)	
2	26 (51.0%)	23 (45.1%)	
3	5 (9.8%)	6 (11.8%)	
Duration (min)	105.0 (90.0–130.0)	100.0 (85.0–130.0)	0.09 (− 0.30, 0.48)

Values are number (proportion), mean (standard deviation), or median (interquartile range). *: it means standardized difference in means or proportions. BMI, body mass index; ASA, American Society of Anesthesiologists; CI, confidence interval

**Fig. 4:**
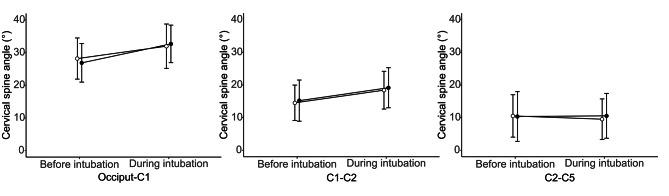
Comparison of cervical spine angles before and during videolaryngoscopic intubation between two glottis exposure methods

Cervical spine movement during videolaryngoscopic intubation was significantly smaller at the occiput–C1 in the direct epiglottis elevation group than in the indirect epiglottis elevation group (3.9 [4.0] vs. 5.8 [3.4] °, difference in means [98.33% CI] − 1.9 [− 3.5, − 0.3] °, P = 0.011), whereas it was not significantly different at the C1–C2 and C2–C5 between the two groups (Table 2). Cervical spine angles before and during videolaryngoscopic intubation at the occiput–C1, C1–C2, and C2–C5 showed no significant differences between the two groups (Fig. 4). In multiple linear regression analysis, all aforementioned variables with significant imbalance between the two groups were not fit to the optimal models for cervical spine movement during videolaryngoscopic intubation at the occiput–C1, C1–C2, and C2–C5. The direct epiglottis elevation group (vs. the indirect epiglottis elevation group) were fit to the optimal model for cervical spine movement during videolaryngoscopic intubation only at the occiput–C1 (R^2^ = 0.054, β = 0.251, P = 0.011).

**Table 2:** Comparisons of cervical spine movement during videolaryngoscopic intubation between direct and indirect epiglottis elevation groups

Variable	Direct epiglottis elevation group (n = 51)	Indirect epiglottis elevation group (n = 51)	Difference in means (98.33% CI)	P value
Cervical spine movement during videolaryngoscopic intubation (°)				
Occiput–C1	3.9^a^ (4.0)	5.8 (3.4)	−1.9 (− 3.5, − 0.3)	0.011
C1–C2	3.8^a^ (4.3)	3.9 (4.1)	−0.1 (− 1.9, 1.8)	0.930
C2–C5	−1.1^a^ (5.0)	0.2 (5.6)	−1.4 (− 3.7, 1.0)	0.198
Cervical spine angle before videolaryngoscopic intubation (°)				
Occiput–C1	28.2 (6.3)	26.9 (5.9)	1.3 (− 1.4, 4.0)	0.280
C1–C2	14.5 (5.4)	15.2 (6.3)	−0.7 (− 3.3, 1.9)	0.564
C2–C5	10.5 (6.5)	10.3 (7.6)	0.2 (− 3.0, 3.3)	0.902
Cervical spine angle during videolaryngoscopic intubation (°)				
Occiput–C1	32.0 (6.8)	32.7 (5.7)	−0.7 (− 3.5, 2.1)	0.573
C1–C2	18.4 (5.8)	19.1 (6.1)	−0.7 (− 3.4, 1.9)	0.527
C2–C5	9.5 (6.2)	10.5 (6.9)	−1.0 (− 4.0, 1.9)	0.426

Values are mean (standard deviation). ^a^: the difference of 0.1 from the computed cervical spine movement between cervical spine angles before and during videolaryngoscopic intubation in this table is due to rounding to two decimal places. CI, confidence interval

A percentage of glottic opening score of 50% was achieved in all patients and all intubations were successful on the first attempt (Table 3). Intubation time was significantly longer in the direct epiglottis elevation group (29.0 [24.0–35.0] vs. 22.0 [18.0–27.0] s, median difference [95% CI] 7.0 [4.0, 10.0] s, P < 0.001). The incidence of bleeding, hoarseness, and sore throat was 7.8%, 43.1%, and 80.4% in the direct epiglottis elevation group, respectively, and 22.5%, 45.1%, and 68.6% in the indirect epiglottis elevation group, respectively.

**Table 3:** Comparison of intubation performance and intubation-related airway complications between direct and indirect epiglottis elevation groups

Variable	Direct epiglottis elevation group (n = 51)	Indirect epiglottis elevation group (n = 51)	Median difference (95% CI)	P value
Intubation performance				
Successful intubation on first attempt	51 (100.0%)	51 (100.0%)		
Intubation time (s)	29.0 (24.0–35.0)	22.0 (18.0–27.0)	7.0 (4.0, 10.0)	< 0.001
Percentage of glottis opening score (%)	50.0 (50.0–50.0)	50.0 (50.0–50.0)	0.0 (0.0, 0.0)	1.000
Intubation-related airway complications	44 (86.3%)	39 (76.5%)		
Bleeding	9 (17.6%)	13 (22.5%)		
Blood in oral cavity	4 (7.8%)	8 (15.7%)		
Blood staining on tracheal tube	9 (17.6%)	13 (25.5%)		
Hoarseness	22 (43.1%)	23 (45.1%)		
1 h after intervention	20 (39.2%)	22 (43.1%)		
24 h after intervention	9 (17.6%)	10 (19.6%)		
Sore throat	41 (80.4%)	35 (68.6%)		
1 h after intervention	41 (80.4%)	35 (68.6%)		
Severity*	4.0 (1.0–6.0)	3.0 (0.0–5.0)	0.5 (0.0, 2.0)	0.268
24 h after intervention	30 (58.8%)	21 (41.2%)		
Severity*	1.0 (0.0–2.0)	0.0 (0.0–2.0)	0.0 (0.0, 1.0)	0.317

Values are number (proportion) or median (interquartile range). *: it was assessed using the numeric rating scale (0 for no pain and 10 for the worst imaginable pain). CI, confidence interval

## Discussion

It is clinically important to minimize cervical spine movement during intubation in patients with cervical spine instability. This randomized controlled trial investigated the effects of the two glottis exposure methods on cervical spine movement during videolaryngoscopic intubation in patients under manual in-line stabilization. Direct epiglottis elevation yielded approximately one-third smaller cervical spine movement during videolaryngoscopic intubation at the occiput–C1 than indirect epiglottis elevation in such patients, which was statistically significant.

This difference in cervical spine movement is thought to be because direct epiglottis elevation requires less lifting force than indirect epiglottis elevation to obtain the same degree of glottis exposure. When performing laryngoscopic intubation through indirect epiglottis elevation, a certain amount of lifting force is needed to visualize the glottis; this force can be transmitted to the cervical spine, resulting in cervical spine movement during laryngoscopic intubation [15]. In addition, cervical spine movement during laryngoscopic intubation is known to increase with the force applied to the laryngoscopic blade [15]. On the other hand, in previous studies, direct epiglottis elevation, compared to indirect epiglottis elevation, has been proven to improve glottis exposure during intubation using videolaryngoscopes as well as direct laryngoscopes [20, 21, 26, 27]. In particular, direct epiglottis elevation has a great advantage in glottis exposure in patients with a floppy epiglottis or a large tongue [20]. In this study, the glottis could easily be revealed with minimal lifting force, once the epiglottis was successfully held by the blade tip in the direct epiglottis elevation group. However, in the indirect epiglottis elevation group, more lifting force was often required to elevate the epiglottis indirectly, despite the fact that videolaryngoscopes, especially with hyperangulated blades, generally need less force applied to the laryngoscopic blade to expose the glottis than direct laryngoscopes [15].

In this study, intubation time was significantly longer in the direct epiglottis elevation group; this could be attributed to the greater amount of time required to properly position the blade tip in the direct epiglottis elevation group. In the indirect epiglottis elevation group, the blade tip could easily be placed in the vallecula by inserting it along the tongue. On the other hand, direct epiglottis elevation required additional time for positioning of the blade tip below the epiglottis. In particular, it was difficult to apply direct epiglottis elevation in patients with a short epiglottis, because such epiglottis frequently slipped out from the blade tip. Another reason for the longer intubation time in the direct epiglottis elevation group could be more difficult insertion of the tracheal tube into the glottis. In the indirect epiglottis elevation group, the blade tip was above the epiglottis, and the tracheal tube passed below the epiglottis without any interference between them. However, in the direct epiglottis elevation group, the blade tip was placed just in front of the glottis, potentially contributing to more difficult insertion of the tracheal tube into the glottis in a few patients. Long intubation times in the direct epiglottis elevation group potentially increase the risk of complications including desaturation and aspiration, especially in patients with cervical spine instability. Nevertheless, this increased risk does not appear to be clinically significant, as the median difference in intubation time between the two groups was only 7 s and no patient showed desaturation below 90%. Furthermore, we speculated that the duration of significant cervical spine movement, which is thought to occur during glottis exposure, would have been comparable between the two groups, because the time to optimal positioning of the blade tip mainly contributed to this difference in intubation time.

Several studies have compared direct and indirect epiglottis elevations during laryngoscopic intubation, but none have investigated their safety [21, 26, 27]. In this study, in contrast with our expectation that there might be more airway complications due to more videolaryngoscopic manipulation for proper positioning of the blade tip in the direct epiglottis elevation group, it was difficult to find a clear trend of more intubation-related airway complications in the direct epiglottis elevation group. On the other hand, special attention should be paid to hemodynamic instability, such as hypotension, bradycardia, and asystole, when applying direct epiglottis elevation. The posterior surface of the epiglottis and laryngeal inlet are dominantly innervated by the vagus nerve; thus, elevating the epiglottis directly with the laryngoscopic blade can cause vagal activation [28] In this study, hemodynamic instability as a result of vagal activation by videolaryngoscopic manipulation was not observed in the direct epiglottis elevation group. This is thought to be due to blocking of vagal activation by applying lidocaine spray on the upper airway, including the epiglottis and glottis.

There were several limitations to this study. First, this study may have been underpowered to determine statistical significance of differences in the incidence of bleeding, hoarseness, and sore throat due to the relatively small sample size. Thus, the statistical results of these outcome measures were not presented. Second, the anesthesiologist performing videolaryngoscopic intubation could not be blinded to the group assignment; thus, this could introduce a potential bias. Third, videolaryngoscopic intubation was performed using C-MAC® D-blade by a skilled anesthesiologist in patients with normal upper airway and cervical spine under manual in-line stabilization; thus, it may be difficult to apply our findings to different intubation circumstances such as operators not proficient in videolaryngoscopic intubation, patients with abnormal upper airway or cervical spine, patients whose cervical spine is not stabilized, videolaryngoscopes with different types of blades or direct laryngoscopes. Fourth, lateral cervical spine radiographs were taken only once both before and during videolaryngoscopic intubation to minimize radiation hazard; there may be a limitation in precisely assessing cervical spine movement throughout intubation, including optimal positioning of the blade tip. Fifth, the percentage of glottic opening score during videolaryngoscopic intubation was set to 50% in this study. If the glottis is visualized minimally or maximally, cervical spine movement during videolaryngoscopic intubation might be different, as the lifting force would differ. Sixth, our findings may not be reproducible in difficult intubations, such as failure to achieve a percentage of glottic opening score of 50%, unsuccessful intubation on the first attempt, and application of airway maneuvers, since all intubations were successful on the first attempt achieving a percentage of glottic opening score of 50% without airway maneuvers in this study. Seventh, although there has been a common perception that small amount and short duration of cervical spine movement during intubation are safe in patients with cervical spine instability, both the actual amount and duration of cervical spine movement during intubation, which lead to neurological complications, have not yet been fully elucidated. Therefore, the clinical significance of the difference in cervical spine movement during videolaryngoscopic intubation at the occiput–C1 between the two groups is uncertain. Further studies are needed to determine the relationship between neurological complications and the amount and duration of cervical spine movement during intubation. Lastly, the mechanism of the difference in cervical spine movement during videolaryngoscopic intubation according to the glottis exposure method has not been proven, because none of the forces applied to the videolaryngoscopic blade, upper airway, or cervical spine was measured directly in this study.

## Conclusions

Direct epiglottis elevation led to statistically significantly smaller cervical spine movement during videolaryngoscopic intubation at the occiput–C1 than indirect epiglottis elevation while maintaining the same degree of glottis exposure using C-MAC® D-blade in patients under manual in-line stabilization. These findings suggest that direct epiglottis elevation could be considered for videolaryngoscopic intubation in patients with known or suspected instability at the occiput–C1.

## Data Availability

The datasets used and/or analyzed during the current study are available from the corresponding author on reasonable request.
